# Clinical Heterogeneity Associated with *MYO7A* Variants Relies on Affected Domains

**DOI:** 10.3390/biomedicines10040798

**Published:** 2022-03-29

**Authors:** Sun Young Joo, Gina Na, Jung Ah Kim, Jee Eun Yoo, Da Hye Kim, Se Jin Kim, Seung Hyun Jang, Seyoung Yu, Hye-Youn Kim, Jae Young Choi, Heon Yung Gee, Jinsei Jung

**Affiliations:** 1 Department of Pharmacology, Graduate School of Medical Science, Brain Korea 21 Project, Yonsei University College of Medicine, Seoul 03722, Korea; kin2844@yuhs.ac (S.Y.J.); wjddk97@yuhs.ac (J.A.K.); tpwls777@yuhs.ac (S.J.K.); tkdnsel93@yuhs.ac (S.H.J.); goldsy420@yuhs.ac (S.Y.); hyeyounkim@yuhs.ac (H.-Y.K.); 2 Department of Otorhinolaryngology, Ilsan Paik Hospital, Inje University College of Medicine, Goyang 10380, Korea; kalosophiana@gmail.com; 3 Department of Otorhinolaryngology, Yonsei University College of Medicine, Seoul 03722, Korea; yoojeeeun1217@yuhs.ac (J.E.Y.); dhk77@yuhs.ac (D.H.K.); jychoi@yuhs.ac (J.Y.C.)

**Keywords:** *MYO7A*, DFNA11, autosomal dominant hearing loss, post-lingual hearing loss

## Abstract

Autosomal dominant hearing loss (ADHL) manifests as an adult-onset disease or a progressive disease. *MYO7A* variants are associated with DFNA11, a subtype of ADHL. Here, we examined the role and genotype–phenotype correlation of *MYO7A* in ADHL. Enrolled families suspected of having post-lingual sensorineural hearing loss were selected for exome sequencing. Mutational alleles in *MYO7A* were identified according to ACMG guidelines. Segregation analysis was performed to examine whether pathogenic variants segregated with affected status of families. All identified pathogenic variants were evaluated for a phenotype–genotype correlation. *MYO7A* variants were detected in 4.7% of post-lingual families, and 12 of 14 families were multiplex. Five potentially pathogenic missense variants were identified. Fourteen variants causing autosomal dominant deafness were clustered in motor and MyTH4 domains of MYO7A protein. Missense variants in the motor domain caused late onset of hearing loss with ascending tendency. A severe audiological phenotype was apparent in individuals carrying tail domain variants. We report two new pathogenic variants responsible for DFNA11 in the Korean ADHL population. Dominant pathogenic variants of *MYO7A* occur frequently in motor and MyTH4 domains. Audiological differences among individuals correspond to specific domains which contain the variants. Therefore, appropriate rehabilitation is needed, particularly for patients with late-onset familial hearing loss.

## 1. Introduction

Nonsyndromic hereditary hearing loss (HL) is caused by 121 genes, of which 54 result in post-lingual sensorineural hearing loss, as of February 2021 [1,2]. More than ten of these genes are both autosomal dominant (DFNA) and recessive (DFNB). Among them, *MYO7A* mutation causes nonsyndromic hearing loss DFNA11 (OMIM #601317), DFNB2 (OMIM #600060), and syndromic hearing loss Usher syndrome type 1 B (USH1B, OMIM #276900), characterized by sensorineural hearing loss, retinitis pigmentosa, and variable vestibular areflexia [3].

*MYO7A* (OMIM #276903), located on chromosome 11q13.5, encodes unconventional myosin VIIa. Various alternative splicing result in short isoforms, and the largest is predicted to be 254 kDa, consisting of 2215 amino acids. *MYO7A* consists of 49 exons and encodes a motor domain (amino acids 65–741) at the N-terminus, which contains an ATP and actin-binding site, followed by the neck domain (amino acid 745–857), containing five isoleucine-glutamine (IQ) motifs as binding sites for other molecules, and a single α-helix (SAH) domain (amino acids 858–935) contributing to the extension of a lever arm length. The tail domain contains two myosin tail homology 4 (MyTH4) domains (amino acids 1017–1253 and 1747–1895), two band 4.1-ezrin-radixin-moesin (FERM) domains (amino acids 1258-1602 and 190–2205), and an SH3 domain (amino acids 1603–1672) [4]. The tail domain regulates MYO7A movement by bending back towards the head-neck domain [5,6]. Although MYO7A has a coiled-coil region between the SAH and tail domains, it is regarded as a monomer [6,7].

MYO7A is expressed in the retina, lungs, testes, kidneys, and outer and inner hair cells of the cochlea [4]. In hair cells, MYO7A was discovered in the stereocilia bundles, cuticular plate, pericuticular necklace, and cell bodies [8]. MYO7A is concentrated at the upper tip-link density near the intracellular domain of CDH23 and binds to USH1G (SANS) and USH1C (Harmonin) using the MyTH4-FERM domain [9,10,11]. It plays an essential role in mechano-electric transduction (MET) and in slow adaptation as a tip-link tension motor [12]. Although the molecular and physiological functions of MYO7A have been revealed, its clinical heterogeneity is a hurdle. Therefore, the present study aims to investigate the genetic prevalence of MYO7A in a cohort of patients with post-lingual sensorineural hearing loss and genotype-phenotype correlation.

## 2. Materials and Methods

### 2.1. Subjects

The Institutional Review Board of the authors’ institute approved this study (IRB number: 4-2015-0659). A total of 318 individuals (156 males and 162 females) from 300 unrelated Korean families with at least one proband diagnosed with post-lingual sensorineural hearing loss were included, after obtaining informed consent.

### 2.2. Clinical Evaluation

Otoscopy, tympanometry, and pure-tone audiometry (PTA) were performed for audiological evaluations. Hearing loss was categorized as mild (26–40 dBHL), moderate (41–55 dBHL), moderate to severe (56–70 dBHL), severe (71–90 dBHL), and profound (>90 dBHL) based on the average PTA threshold across the four frequencies (500, 1000, 2000, and 4000 Hz). The audiogram configuration was characterized as ascending when thresholds for high frequencies (2000 and 4000 Hz) were less by 25 dB than those for low frequencies (250 and 500 Hz), down sloping when low frequencies were less by 25 dB than high frequencies, and flat when the difference between high and low frequencies was within 25 dB. A bithermal water caloric test was performed to evaluate vestibular function. All the affected individuals denied having ophthalmologic symptoms.

### 2.3. Mutational Analysis of MYO7A

Whole exome sequencing (WES) and variant filtering were performed using the SureSelect V5 enrichment capture kit (Agilent Technologies, Santa Clara, CA, USA) and Illumina HiSeq 2500, as described previously [13]. Briefly, sequence reads were mapped to the human reference genome assembly (NCBI build 3/hg19) using CLC Genomic Workbench (version 9.5.3) software (Qiagen, Toronto, ON, Canada). All variants with a minimum coverage of two were used. Variants were called using Basic Variant Caller of CLC Genomic Workbench and annotated. Filtered variants were evaluated according to the guidelines of the American College of Medical Genetics and Genomics (ACMG). Segregation analysis was performed by Sanger sequencing with DNA samples of additional members from the families.

### 2.4. Copy-Number Variant (CNV) Analysis

To set aside the possibility of patients diagnosed with large exonic deletions or duplications in known hearing loss genes, CNV analysis was performed on paired-end WES data using EXCAVATOR version 2.226 (https://www.ncbi.nlm.nih.gov/pmc/articles/PMC4053953/; accessed on 21 August 2020) and ExomeDepth version 1.1.1027 tools (https://pubmed.ncbi.nlm.nih.gov/22942019/; accessed on 16 July 2020) with default settings, as previously described [13]. The GRCh37/hg19 database was used as the reference assembly to calculate GC content. The WES dataset of 32 audiometrically proven normal individuals was used as a control for CNV analysis.

### 2.5. Variants Database Review in Patients with MYO7A Variants

We searched “*MYO7A*” in three genomic variants databases, ClinVar (https://www.ncbi.nlm.nih.gov/clinvar/; accessed on 10 September 2021.), Deafness Variation Database (DVD, https://deafnessvariationdatabase.org/sources; accessed on 10 September 2021), and Human Gene Mutation Database (HGMD, http://www.hgmd.cf.ac.uk/ac/index.php; accessed on 10 September 2021). We retrieved variants listed as “pathogenic” as of October 2020. We included variants that contributed to non-syndromic autosomal dominant hearing loss (NSADHL) or DFNA11, satisfying the AD cutoff (MAF < 0.0005 and CADD score > 20). Variants linked only to Usher syndrome and DFNB2 were excluded from the analysis. All these variants were then mapped to the longest isoform of MYO7A (NM000260) using lollipopPlot2 of maftools using R script. To compare the location of dominant *MYO7A* variants with that of recessive variants, we extracted a list of pathogenic DFNB2 variants from DVD with the same AD cutoff (MAF < 0.0005 and CADD score > 20). In addition, we searched “DFNA11” in PubMed to identify audiological phenotypes of affected individuals, as of January 2022.

## 3. Results

### 3.1. MYO7A Variants Detected in Yonsei University Hearing Loss (YUHL) Cohort

**Figure 1:**
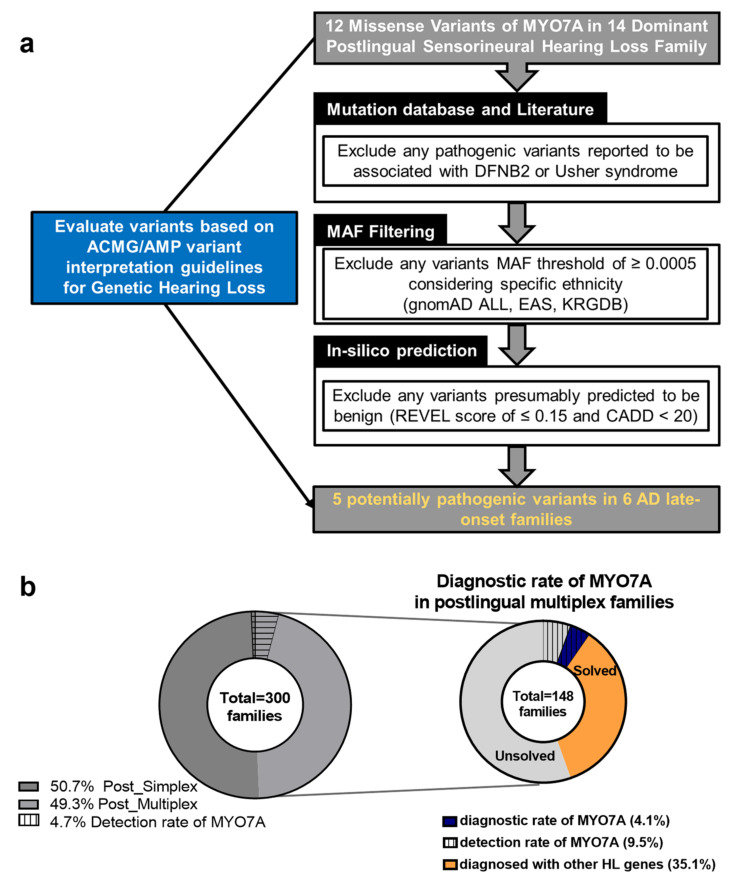
Diagnostic rate of *MYO7A* variants in families with multiplex post-lingual HL and filtering process based on ACMG guideline. (**a**) The pedigree configuration of 300 post-lingual HL families who underwent WES is on the right side. Detection rate of *MYO7A* variants marked with dashed line either in total post-lingual HL families (n = 300) or in post-lingual multiplex families (n = 148). All individuals genetically diagnosed with DFNA11 variants are members of unrelated post-lingual multiplex families, showing 4.1% of diagnostic rate (6/148 families) on the left colored in navy. (**b**) Filtering process utilized in evaluating all 12 detected *MYO7A* variants. After major three steps indicated above, 5 of 12 variants were identified as potential genetic cause of six probands with post-lingual hearing impairment suspected of dominant inheritance.

To select potentially pathogenic variants responsible for post-lingual sensorineural hearing loss related to DFNA11, we conducted stepwise filtering based on internal criteria (Figure 1b). First, we excluded likely pathogenic or pathogenic variants involved in DFNB2. As all missense variants of *MYO7A* detected in our cohort were single heterozygous variants, we did not include pathogenic variants that caused hearing loss in an autosomal recessive manner. Second, we excluded variants with an MAF threshold higher than 0.0005 for autosomal dominant inheritance. Taking specific ethnicity into consideration, we filtered out relatively frequent variants (MAF < 0.0005) in both the East Asian and Korean populations (referred to as gnomAD EAS, KRGDB). (Table 1) [14,15,16,17]. We filtered the remaining variants predicted to be benign based on a REVEL score of 0.15 and a CADD score of 20. We also assessed detected variants of *MYO7A* by observing the ACMG/AMP hearing loss variant guidelines specified by the Clingen hearing loss expert panel. We utilized the variant interpretation platform for genetic hearing loss (VIP-HL), which is a semi-automated and integrated online tool for classifying variants contributing to genetic hearing loss [18]. For the final interpretation of variants, we proceeded with segregation analysis and literature search to add PP1 and PS1 criteria to the VIP-HL interpretation (asterisk mark in “classification” column of Table 1).

Among the 300 families with sensorineural hearing loss after the first decade of life in the YUHL cohort, we detected 12 heterozygous missense variants of *MYO7A* in 14 unrelated families. Five potentially pathogenic variants were identified in six multiplex families after variant evaluation. Therefore, the genetic diagnostic rate of *MYO7A* variants was 2.0% (6/300 families) in total post-lingual cases and 4.1% (6/148 families) in multiplex post-lingual cases (Figure 1b).

**Figure 2:**
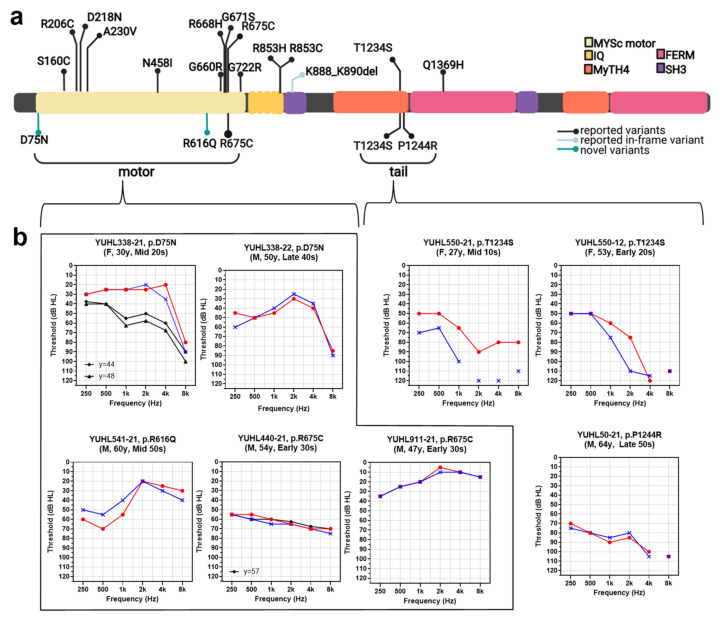
Variants of *MYO7A* identified in this study and audiogram of individuals diagnosed with DFNA11. (**a**) 15 DFNA11 variants previously reported (upper side of MYO7A functional regions) and five variants identified in this study (lower side). Among 18 variants of MYO7A, 12 were located at the motor region (MYSc; head domain) and three (p.R853H, p.R853C and p.K888_K890del) in the neck region. Including two other variants identified in this study, three variants were located at tail region, two (p.T1234S and p.P1244R) at MyTH4 domain, and one at FERM domain. (**b**) Onset-informed audiograms of probands of all DNFA11 families in YUHL cohort. All the unrelated DFNA11 families show phenotypic diversity of MYO7A variants. However, degree of hearing loss is clinically different; patients with variants at motor regions show mild to moderate hearing loss. Individuals with tail region variants show severe hearing loss. Red indicates right side PTA threshold of patients, while line in blue means PTA on the left side.

All variants identified in our study were missense variants (Table 1 and Figure 2a), including c.223G>A (p.Asp75Asn), c.1847G>A (p.Arg616Gln), c.2023C>T (p.Arg675Cys) [19], c.3701C>G (p.Thr1234Ser) [20], and c.3731C>G (p.Pro1244Arg) [21]. All five variants were assigned to one or two pathogenic components using the VIP-HL platform. Three of them (p.Arg675Cys, p.Thr1234Ser, and p.Pro1244Arg) were already reported in the Deafness Variation Database (DVD) as pathogenic in causing ADSNHL (p.Arg675Cys and p.Thr1234Ser) or Usher syndrome with unconfirmed segregation analysis (p.Pro1244Arg). Considering all interpretation criteria, these three variants were classified as “likely pathogenic” according to the ACMG/AMP guidelines. The other two *MYO7A* variants, c.223G>A (p.Asp75Asn) and c.1847G>A (p.Arg616Gln), were novel in their link to ADNSHL. They had evolutionarily conserved altered residues and fulfilled at least one PM criterion (Table 1 and Figure S1). These two variants were rare in the population database (PM2). Segregation of p.Asp75Asn variant in the affected sibling mother (YUHL 338-22) was confirmed by Sanger sequencing (Figures S2 and S3). The p.Arg616Glu variant found in the YUHL541 family was segregated in the unaffected son (YUHL541-31) with normal PTA, suffering from occasional dizziness and difficulties in communication. YUHL541-31 was 34 years old, younger than the disease onset age of YUHL541-21 (the mid-50s).

Three of the five identified variants resided in the N-terminal myosin motor domain of MYO7A. With overlapping missense variants of p.Arg675Cys found in both YUHL440 and YUHL911, 14 individuals (YUHL338-21, 338-22, and 338-31; YUHL440-21, 440-23, 440-24, 440-25, 440-32, and 440-33; YUHL541-21 and 541-31; and YUHL911-21, 911-12, and 911-22) from four unrelated families (YUHL338, YUHL440, YUHL541, and YUHL911) harbored missense variants, which led to a single amino acid substitution in the myosin motor domain (Figure 2a). Three affected individuals (YUHL50-21, YUHL550-21, and 550-12) from two unrelated families (YUHL50 and 550) carried two closely located missense alleles affecting the tail (1st MyTH4) domain.

### 3.2. Clinical Phenotype in Korean DFNA11 Population

We described the clinical phenotypes of 8 individuals from 6 unrelated families (Table 2 and Figure 2b). All individuals showed post-lingual onset of HL, and the age of onset ranged from the second to sixth decades. YUHL 440-21 and YUHL 911-21 shared same missense variant c.2023C>T (p.Arg675Cys), in the motor domain. HL began in the early 30s. In our cohort, YUHL550-21 carrying the tail domain variant had the earliest onset of HL during teenage, while her brother (YUHL550-22) noticed HL in his late 40s. Although the audiometric configurations varied, they tended to depend on the affected motor and MyTH4 domains (Figure S4). Five individuals bearing motor domain variants had different audiograms; one of the earliest p.Asp75Asn variants affected high frequency-dependent HL albeit concomitant low frequency dependence of sibling. p.Arg616Glu and one of p.Arg675Cys were low-frequency dependent. One of the p.Arg675Cys mutants exhibited a flat configuration. The tail domain variants exhibited a down sloping pattern. The anamnestic hearing test was available in YUHL338-21 carrying early motor domain variant (p.Asp75Asn). Initially, HL affected higher frequencies and gradually progressed to all frequencies. The individual showed rapid progression of 35 dB over 18 years, and also admitted explicit noise exposure as a result of hard rock mania since adolescence. Therefore, noise exposure could have provided synergic effects on hearing loss progression. Regarding the severity of hearing loss, three individuals from two families (YUHL50-21, 550-12, and 550-21) carrying MyTH4 domain variants presented severe HL, whereas the others showed mild to moderate HL. YUHL50-21 received a unilateral cochlear implant and used a hearing aid on the other side. All others wore hearing aids for hearing rehabilitation, except YUHL541-21 and YUHL 911-21. Both YUHL541-21 and YUHL50-21 attested to frequent vertigo spells since their 50s-60s, and the caloric test revealed right unilateral vestibulopathy (YUHL50-21) and no weakness (YUHL541-21). Other individuals also experienced occasional mild dizziness, but the characteristics were not regarded as originating from vestibular areflexia. None of the affected individuals had ophthalmologic symptoms, such as difficulty seeing at night and loss of side vision suspected to involve the retina.

### 3.3. Variants Database Review in Individuals with MYO7A Variants

Pathogenic *MYO7A* variants were collected from three databases: DVD, ClinVar, and HGMD. Except for *MYO7A* variants that only caused Usher1B or DFNB2, variants related to DFNA11 and fulfilling the AD cutoff were included. We selected 14 missense variants and one in-frame deletion variant, that cause non-syndromic hearing loss in an autosomal dominant manner (Figure 2a; shown on top of the drawing). Two variants, c.652G>A (p.Asp218Asn) and c.689C>T (p.Ala230Val) were evaluated as pathogenic in all three databases [22,23,24]. Among the 15 confirmed DFNA variants, 10 were located in the N-terminal myosin motor domain, three were in the IQ motif, one was in the MyTH4 domain, and one was in the FERM domain (Figure 2a and Table 3). Interestingly, distribution of dominant variants was more enriched in specific domains than that of pathogenic recessive *MYO7A* variants (Figure S5). More than 66% (10 of 15) of pathogenic DFNA 11 variants from the database resided in the N-terminal Myosin motor domain, showing similar distribution with our cohort. Combined with two novel variants from our cohort, the motor domain contained 66.7% (12 of 18) of DFNA11-related variants. This may indicate a domain with mutational hotspots.

**Figure 3:**
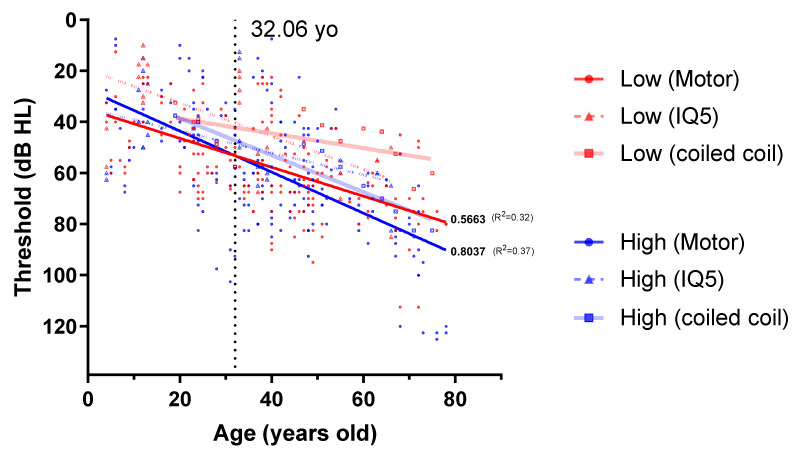
Changes in low and high frequency hearing thresholds by age for MYO7A variants. The hearing thresholds from literature with MYO7A variants are represented with red (low frequency: 250~500 Hz) and blue (high frequency: 2000~4000 Hz). Lines correspond to linear regression of best fit. Pure tone audiometry (PTA) results of 70 individuals with motor domain, 12 with IQ domain, and 5 with coiled-coil domain variants were denoted as circles with line, circles with dashed line and rectangles with transparent line, respectively. Unlike in other regions of MYO7A, variants in the motor domain showed low-frequency dominant hearing loss, particularly until the fourth decade (32.06 years old). PTA results of motor domain variants showed more rapid progression in high frequency deterioration like that of the other variants in functional domains, such as IQ or SAH domain, from the fourth decade (R^2^: 0.37).

Of the 15 reported pathogenic variants, audiological phenotypes in 11 families with eight missense variants and one in-frame deletion variant were obtained from PubMed (Table 3 and Table 4). Among the 11 families, three were Caucasian and the others were East Asian. The majority of the variants existed in the motor domain (70 individuals, 183 audiograms), one in the IQ domain (12 individuals, 24 audiograms), and one in the coiled-coil domain (five individuals, 13 audiograms), with none observed in the tail region. Along with audiological reports, average threshold of low and high frequencies was depicted as a function of age by linear regression, according to affected variants (Figure 3). Apparently, low-frequency dependent HL tended to be dominant until the fourth decade (32.06 years old) when bearing motor variants. Furthermore, the slope at high frequency was stiffer than that at low frequency; in other words, high-frequency deterioration might be more rapid. In contrast, high frequency-dependent HL was prominent across all ages in other variants. One variant (c.689C>T; p.Ala230Val) was detected in both Italian and Japanese ethnicities with an inconsistent phenotype [22,24]. The Italian variant showed high-frequency dominant HL, and few of the affected family members had vestibular areflexia. The Japanese variant was mid-frequency dominant and did not encompass the vestibule. Another motor domain variant (c.2011G>A) was segregated from two large Chinese families [23,25]. In both families, HL started at low frequencies in early adulthood and progressed to a flat configuration in their 40s. The other seven variants were detected, one from each of the seven families. Vestibular dysfunction was observed in only two families: Dutch and Italian. Ocular symptoms or dysfunction were never reported in patients with DFNA11.

**Table 3:** Clinically reported DFNA11 affected families in the literatures.

Ethnicity	Nucleotide Change	Amino Acid Change	Domain	Audiogram Configuration ^a^ (Number of Patients)	Onset (Decade)	Annual Threshold Increase (Cross-Sectional)	Vestibular Symptoms	Retinal Degeneration	Reference
Chinese	c.616C>T	p.Arg206Cys	Motor	Down sloping (2)	3rd–4th	n/a	none	none	[26]
				Flat (1)					
Chinese	c.652G>A	p.Asp218Asn	Motor	Down sloping (4)	3rd–5th	modest	none	none	[23]
				Flat (1)					(DX-J033)
Italian	c.689C>T	p.Ala230Val	Motor	flat (5)	1st	n/a	3	none	[22]
				Down sloping (1)					
Japanese	c.689C>T	p.Ala230Val	Motor	Down sloping (1)	1st	n/a	none	none	[24]
Dutch	c.1373A>T	p.Asn458Ile	Motor	Down sloping (5)	1st–2nd	0.3–0.9	4	none	[27,28]
				Ascending (4)					
Chinese	c.2003G>A	p.Arg668His	Motor	Flat (9)	2nd–5th	modest	n/a	n/a	[29]
Chinese	c.2011G>A	p.Gly671Ser	Motor	Ascending (3)	2nd–4th	modest	none	none	[23]
				Flat (2)					(HB-S037)
Chinese	c.2011G>A	p.Gly671Ser	Motor	Flat (14)	2nd–5th	modest	none	none	[25]
				Ascending (3)					(Z029)
				Normal (3)					
				Down sloping (1)					
American	c.2164G>C	p.Gly722Arg	Motor	Flat (8)	3rd–4th	n/a	none	none	[30]
				Down sloping (3)					
Japanese	c.2558G>A	p.Arg853His	IQ5	Down sloping (7)	1st–4th	0.54–1.03	none	none	[31]
				Flat (5)					
Japanese	c.2662_2670del	p.Lys888_Lys890del	Coiled coil	Flat (3)	2nd	0.41–0.74	none	none	[3,32]
				Down sloping (2)					

^a^ When serial audiograms of one individual were obtainable, the configuration was considered by final hearing at the oldest age.

## 4. Discussion

In the present study, we performed WES on 300 Korean families and six of them were diagnosed with DFNA11. As the Korean DFNA11 population has never been reported, the YUHL cohort incidence could be the first report contributing to 2.0% (6/300 families) of total post-lingual cases and 4.1% (6/148 families) in multiplex post-lingual cases. In addition, we introduced two novel and probable pathogenic variants of DFNA11 and presented a distinctive clinical phenotype of MyTH4 domain variant for the first time. Although *MYO7A* can cause USH1B, DFNB2, and DFNA11, no autosomal recessive inherited variants were found in our cohort. To date, most studies reporting DFNA11 are conducted using linkage analysis with a few large families. Therefore, affected small families or probable sporadic cases are disregarded. In this respect, the clinical spectrum of DFNA11 has scarcely been revealed, and little is known about its global or ethnic incidence rates. Since the first report of a 9 bp in-frame deletion variant in the SAH region of a Japanese family, 12 families worldwide have been reported to date [3,22,23,24,25,26,27,28,29,30,31,32,33,34]. Here, we added six more families with genotypic and phenotypic characteristics of DFNA11. Therefore, this study could make a valuable contribution to expanding the field of hereditary hearing loss.

A c.3701C>G (p.Thr1234Ser) mutation was reported in one Korean subject, which possibly caused compound heterozygous DFNB1 to develop severe sensorineural HL [20]. Due to the fact that the detailed co-segregation data of the subject are insufficient, the potential of this variant to be the founder allele in the Korean population is not clear. Instead, considering that c.2023C>T (p.Arg675Cys) was discovered in two unrelated families, it might be the founder allele. In the Japanese population, c.2023C>T (p.Arg675Cys) and c.3701C>G (p.Thr1234Ser) were also regarded as inherited in an autosomal dominant fashion; however, the clinical phenotype was not shared [19,34].

The audiological configuration, onset, or progression rate represented inter or intrafamilial variability among variants in the motor domain (Table 2 and Table 3). Therefore, *MYO7A* modifier might affect the wild-type promoter allele [35]. In the YUHL cohort, audiological phenotype was distinct in accordance with the affected domain. Missense variants in the motor domain of individuals (except YUHL 338-21, possibly noise-induced HL) were expressed during adult-onset, slowly progressed, and ascended to a flat configuration. In contrast, individuals carrying MyTH4 domain variants showed adult-onset, rapid progression, and a down sloping tendency.

Mouse models with heterogeneous human-like phenotypes have been used to replicate human *MYO7A* variants [12,36,37,38,39]. Several strains exhibited phenotypes similar to those observed in this study. Of the missense alleles in the motor domain, *headbanger* heterozygotes bearing c.531A>T (p.L178F) in exon 6 exhibited early age low-frequency HL and residual hearing at high frequencies [37]. Consistently, morphological disorganization of the hair bundle was prominent in the whole inner hair cell (IHC) and the apical area of outer hair cell (OHC). In addition, *dumbo* heterozygotes with c.2839T>A (p.F947I), a highly conserved residue in the linker region of exon 23, also express similar tonotopic HL [39]. Although strains with missense mutation in the tail domain (2nd MyTH4 and FERM) have been reported, a heterozygous phenotype has not been outlined [36].

Conditional removal of the longest canonical isoform in a mouse model (*Myo7a-ΔC)* resulted in tonotopical loss of *MYO7A* expression in hair cells [12]. In *Myo7a-ΔC* mice, *MYO7A* expression was lost in whole IHC and OHC apical to mid-turn, although hair bundle morphology was normal (P5). In addition, a well-developed hair bundle was markedly disrupted, and gradual HL progressed to a profound level with age (9 weeks), despite near-normal hearing at early ages (P17). Resting open probability (P_o_) was not changed in basal OHC, and was significantly reduced in IHC, in comparison to that of the wild type strain. The remaining isoforms could have completed the development of stereocilia but were insufficient to maintain tip-link tensioning for MET.

Meanwhile, heterozygous *Myo7a^+/−^* mice showed well-preserved IHC in the apical and middle turns, impaired OHC predominantly in the basal turn, and severe hearing loss at medium to high frequencies [40]. Myosin VIIa was distributed not only to the stereocilia but also to the entire length of the hair cell cytoplasm. Therefore, myosin staining has been widely used to distinguish against supporting cells [4,9]. HL in *Myo7a^+/−^* mice was contrary to that with myosin VIIa distribution in stereocilia. Rather, it was similar to age-related hearing loss. Insufficiency of myosin VIIa in the cytoplasm may contribute to cellular dysfunction, which has not yet been elucidated. Myosin VIIa is highly expressed in the retinal pigment epithelium and is known to play a role in positioning melanosomes as lysosome motors [41]. The relationship between lysosomes in hair cell cytoplasm and the cargo-binding function of myosin VIIa is elusive. If cytoplasmic myosin VIIa is related to lysosomal transportation, insufficient myosin VIIa may inhibit the breakdown of autophagosomes and cause autophagy dysfunction, resulting in early cell death and aging of sensory epithelium in the inner ear [42,43].

In conclusion, we report the incidence of DFNA11 in the Korean ADNSHL population and introduce two novel variants of MYO7A. In addition, audiological differences, depending on the affected domain, have been identified. The novel insight into the affected domains might shed light on elucidating the heterogeneous phenotype in DFNA11 to suggest appropriate genetic counseling and rehabilitation.

## Figures and Tables

**Table 1:** Variants in *MYO7A* identified in individuals with autosomal dominant hearing loss by exome sequencing.

Individual	Sex	Age of Onset (Years)	Nucleotide Change ^a^	Amino Acid Change	Exon	Zygosity	Amino Acid Sequence Conservation ^b^	dbSNP ^c^	ESP ^d^	gnomAD ^e^	gnomAD EAS	KRGDB ^f^	Condel ^g^	REVEL ^h^	CADD ^i^	Clinvar	DVD	Classification ^j^	Evaluation
YUHL 338-21 338-22	F M	Mid 20s Late 40s	c.223G>A	p.Asp75Asn	4/49	Het	Danio rerio	ND	ND	ND	ND	ND	Del (0.895)	0.521	32	ND	ND	PM2,	VUS
YUHL 440-21	M	Early 30s	c.2023C>T	p.Arg675Cys	16/49	Het	Danio rerio	rs782459520	ND	0.00002018	0.0001671	0.000454545	Del (0.906)	0.893	29.8	Uncertain Significance	Pathogenic for AD, sporadic HL	PM2, PP3, PS1 *, PP1 *	Likely pathogenic
YUHL 911-21	M	Early 30s																	
YUHL 550-21 550-12	F F	Mid 10s Early 20s	c.3701C>G	p.Thr1234Ser	29/49	Het	Danio rerio	rs775908821	ND	0.00003592	0.0004964	ND	Del (0.847)	0.689	25.6	Uncertain Significance	Likely Pathogenic for ADSNHL	PM2_Supporting, PS1 *,	Likely pathogenic
YUHL 50-21	M	Late 50s	c.3731C>G	p.Pro1244Arg	29/49	Het	Danio rerio	ND	ND	ND	ND	ND	Del (0.935)	0.946	29.8	ND	Pathogenic For Usher Syndrome 1	PM2, PP3, PS1	Likely pathogenic
YUHL 541-21 541-31	F F	Mid 50s -	c.1847G>A	p.Arg616Gln	16/49	Het	Danio rerio	rs782410686	ND	0.00005112	0.00007676	ND	Neu (0.434)	0.291	24.4	ND	Unknown Significance	PM2	VUS

Abbreviations: Ben, benign; Dam, probably damaging; Del, deleterious; Neu, neutral; DC, disease-causing; Tol, tolerated; het, heterozygous in the affected individual; F, female; M, male; ND, no data or DNA not available; Neu, neutral. ^a^ cDNA mutations are numbered according to the human cDNA reference sequence NM_000260.4 (MYO7A); +1 corresponds to A of ATG translation initiation codon. ^b^ Amino acid residue is continually conserved throughout evolution, including the species indicated. ^c^ dbSNP database (http://www.ncbi.nlm.nih.gov/SNP; accessed on 10 September 2021.). ^d^ NHLBI Exome Sequencing Project (http://evs.gs.washington.edu/EVS/). ^e^ gnomAD browser (http://exac.broadinstitute.org/; accessed on 10 September 2021.). ^f^ The Korean Reference Genome Database. ^g^ Condel (http://bbglab.irbbarcelona.org/fannsdb/; accessed on 10 September 2021.). ^h^ REVEL (https://sites.google.com/site/revelgenomics/; accessed on 10 September 2021.). ^i^ Phred-like scores (scaled C scores) on the Combined Annotation Dependent Depletion (http://cadd.gs.washington.edu/home/; accessed on 10 September 2021.). ^j^ Classification of variants based on a combination of internal evaluation and classification by VIP-HL (variant interpretation platform for genetic hearing loss (http://hearing.genetics.bgi.com/; accessed on 10 September 2021.). * Asterisk marks mean variant classification based on segregation analysis and literature search.

**Table 2:** Clinical profiles of individuals with DFNA11 in YUHL cohorts.

Individual	Sex	Age of Initial Test (Years)	Age of Onset (Years)	Vestibular Symptom	Caloric Test	Ophthalmologic Symptom or Exam	Audiogram Configuration	Severity	Auditory Rehabilitation
YUHL 50-21	M	64	Late 50s	Not noticed until the 7th decade of life	Unilateral weakness	No symptom	Flat	Severe	CI(R) + HA(L)
YUHL 338-21	F	30	Mid 20s	Not noticed until the 5th decade of life	NA	No symptom	Flat	Mild	HA(B)
YUHL 338-22	M	50	Late 40s	No vestibular disturbance	NA	No symptom	Flat	Mild	HA(R)
YUHL 440-21	M	54	Early 30s	Intermittent minor vertigo	NA	No symptom	Flat	Moderate	HA(B)
YUHL 541-21	M	60	Mid 50s	Instability when walking Disequilibrium on walking(Normal VFT)	No weakness	Mild visual acuity deterioration but no retinitis pigmentosa-	Ascending	Moderate Mild	None-
YUHL 550-21	F	27	Mid 10s	Intermittent minor vertigo	NA	No symptom	Down-sloping	Severe Profound	Planning CI(L) + HA(R)
YUHL 550-12	F	53	Early 20s	No vestibular disturbance-	NA	No symptom	Down-sloping	Severe	NA
YUHL 911-21	M	41	Early 30s	Intermittent minor vertigo	No weakness	No symptom	Flat	Mild	None

Abbreviations: CI, cochlear implant; HA, hearing aid; NA, not available.

**Table 4:** MYO7A variants associated with DFNA11 in the literatures.

Nucleotide Change ^a^	Amino Acid Change	Exon	Amino Acid Sequence Conservation ^b^	dbSNP ^c^	ESP ^d^	gnomAD ^e^	gnomADEAS	KRGDB ^f^	Condel ^g^	REVEL ^h^	CADD ^i^	Clinvar	DVD	VIP-HL ^j^
c.616C>T	p.Arg206Cys	7/49	Danio rerio	rs782361954	ND	0.00002408	0.0002225	ND	Del (0.911)	0.976	32	uncertain_significance	Pathogenic for DFNA11	PM2_Supporting, PP3
c.652G>A	p.Asp218Asn	7/49	Danio rerio	rs201539845	ND	0.00004634	0	ND	Del (0.765)	0.651	29.9	pathogenic, likely_pathogenic	Likely Pathogenic for DFNA11	PM2_Supporting
c.689C>T	p.Ala230Val	7/49	Danio rerio	rs797044512	ND	ND	ND	ND	Del (0.897)	0.818	29.1	pathogenic, likely_pathogenic	Pathogenic for DFNA11	PM2, PP3
c.1373A>T	p.Asn458Ile	13/49	Danio rerio	rs121965084	ND	0.00001281	0.00005735	ND	Del (0.861)	0.884	26.5	pathogenic	Pathogenic for DFNA11	PM2, PP3
c.2003G>A	p.Arg668His	17/49	Danio rerio	rs368575149	ND	0.000004050	0	ND	Del (0.897)	0.886	31	ND	Pathogenic for DFNA11	PM2, PP3
c.2011G>A	p.Gly671Ser	17/49	Danio rerio	rs387906699	ND	ND	ND	ND	Del (0.935)	0.967	30	uncertain_significance, pathogenic	Pathogenic for DFNA11	PM2, PP3
c.2164G>A	p.Gly722Arg	18/49	Danio rerio	ND	ND	ND	ND	ND	Del (0.945)	0.982	28.3	ND	Pathogenic for DFNA11	PM2, PP3
c.2557C>T	p.Arg853Cys	21/49	Danio rerio	ND	ND	ND	ND	ND	Del (0.867)	0.801	29.1	ND	Pathogenic for DFNA11	PM2, PP3, PM5
c.2558G>A	p.Arg853His	21/49	Danio rerio	rs111033437	ND	0	0	ND	Del (0.897)	0.741	31	uncertain_significance, likely_pathogenic	Likely Pathogenic for DFNA11	PM2, PP3
c.2662_267del	p.Lys888_Lys890del	22/49	Xenopus tropicalis	ND	ND	ND	ND	ND	ND	ND	20.9	ND	Pathogenic for DFNA11	PM2

Abbreviations: Ben, benign; Dam, probably damaging; Del, deleterious; Neu, Neutral; DC, disease-causing; Tol, tolerated; het, heterozygous in affected individual; F, female; M, male; ND, no data or DNA not available; Neu, neutral; PP2, PolyPhen-2 prediction score Humvar; SIFT, Sorting Intolerant from Tolerant. ^a^ cDNA mutations are numbered according to human cDNA reference sequence NM_000260.4 (*MYO7A*); +1 corresponds to the A of the ATG translation initiation codon. ^b^ Amino acid residue is continually conserved throughout evolution, including the species indicated. ^c^ dbSNP database (http://www.ncbi.nlm.nih.gov/SNP; accessed on 10 September 2021). ^d^ NHLBI Exome Sequencing Project (http://evs.gs.washington.edu/EVS/; accessed on 10 September 2021). ^e^ gnomAD browser (http://exac.broadinstitute.org/; accessed on 10 September 2021). ^f^ The Korean Reference Genome Database. ^g^ Condel (http://bbglab.irbbarcelona.org/fannsdb/; accessed on 10 September 2021). ^h^ REVEL (https://sites.google.com/site/revelgenomics/; accessed on 10 September 2021). ^i^ Phred-like scores (scaled C scores) on Combined Annotation Dependent Depletion (http://cadd.gs.washington.edu/home/; accessed on 10 September 2021). ^j^ Variant Interpretation Platform for Genetic Hearing Loss (http://hearing.genetics.bgi.com/; accessed on 10 September 2021).

## Data Availability

The data presented in this study are available on request from the corresponding author. The data are not publicly available due to privacy.

## Supplementary Materials

The following supporting information can be downloaded at: https://www.mdpi.com/article/10.3390/biomedicines10040798/s1, Figure S1: Evolutionary conservation of altered amino acid residue of MYO7A; Figure S2: Pedigrees and variants identified in *MYO7A* in Korean families; Figure S3: Sanger sequencing traces of six families with *MYO7A* variants in YUHL cohort; Figure S4: Audiograms of the eight affected individuals with *MYO7A* variants in YUHL cohort; Figure S5: Distribution of DFNB2-related pathogenic *MYO7A* variants.
