# Supplementary material for: Clinical Heterogeneity Associated with MYO7A Variants Relies on Affected Domains
Source: Biomedicines. 2022 Mar 29;10(4):798. doi: 10.3390/biomedicines10040798 (PMC9028242; doi:10.3390/biomedicines10040798)
Supplement: Supplementary file 1 [file biomedicines-10-00798-s001.zip › biomedicines-1649699-supplementary.pdf]

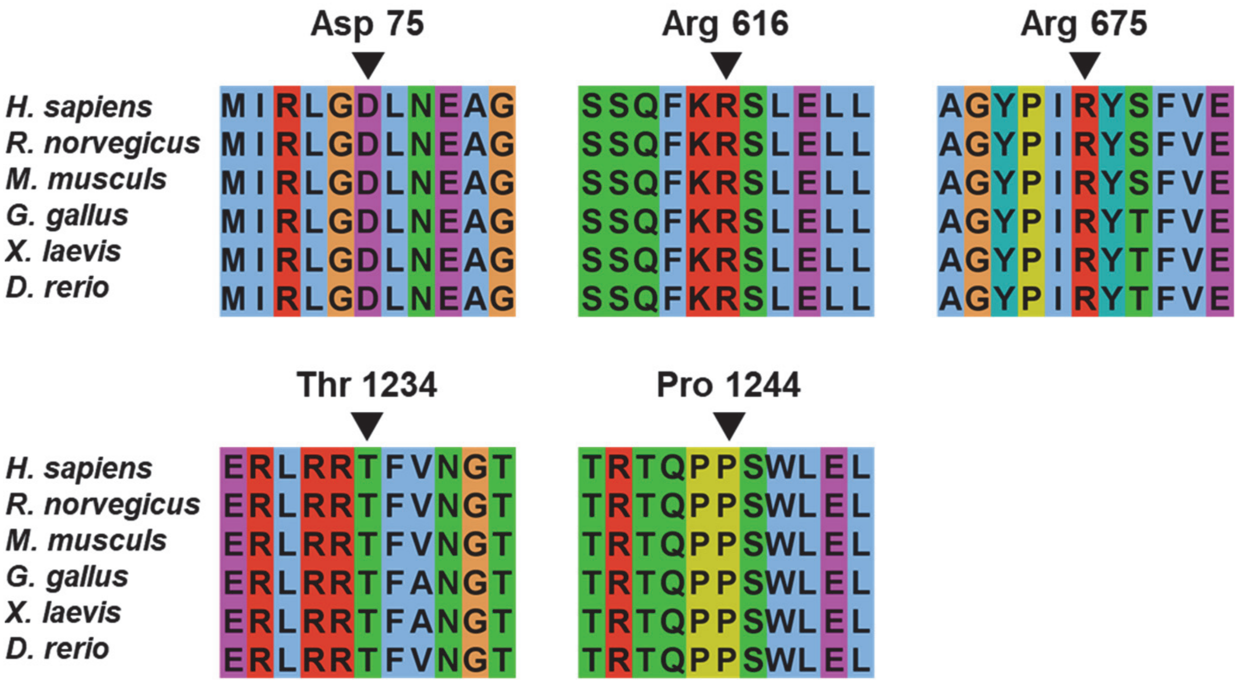

Figure S1. Evolutionary conservation of altered amino acid residue of MYO7A. Amino acid alignment showing conservation of identified residues in MYO7A across different species: *R. norvegicus*, *M. musculus*, *G. gallus*, *X. laevis*, and *D. rerio*.

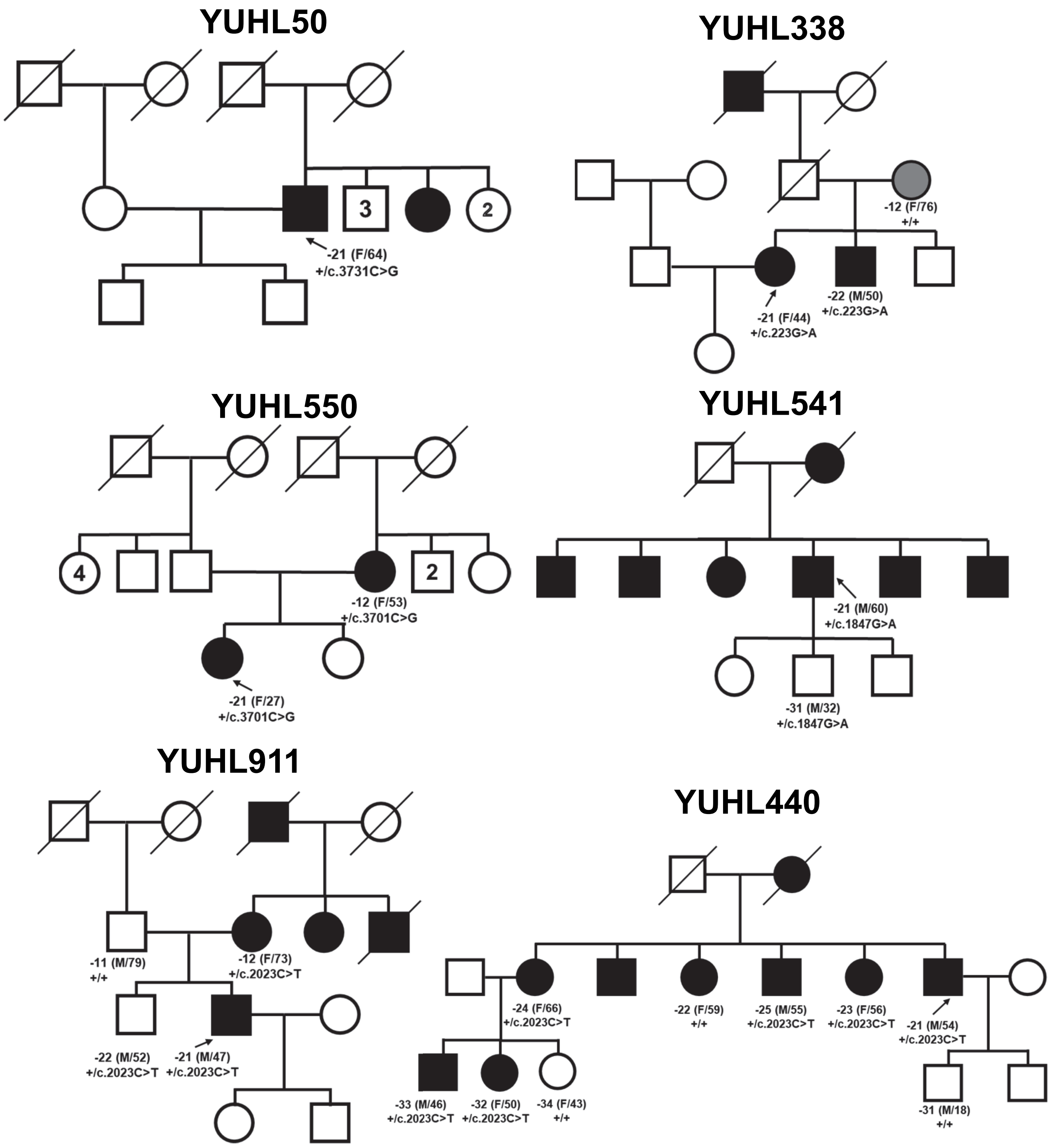

Figure S2. Pedigrees and variants identified in *MYO7A* in Korean families. Circles represent females and Squares males. Filled symbols indicate individuals with HL and clear symbols denotes unaffected individuals. The number in the clear symbols briefly indicates the number of unaffected relatives in families. Grey filled symbols represent affected individuals not due to MYO7A mutations. Probands with WES data were pointed. Genotypes below indicated every individual for which a DNA sample was available for sanger sequencing.

Figure S3. Sanger sequencing traces of six families with *MYO7A* variants in YUHL cohort. Sanger sequencing traces of five variants in *MYO7A* are shown for individuals from six families; YUHL50, YUHL338, YUHL440, YUHL541, YUHL550 and YUHL911. The altered amino acid is written in red. In the case of YUHL50 in which no sample from affected relatives was available, sample of patient with no MYO7A variant at the site was used as control.

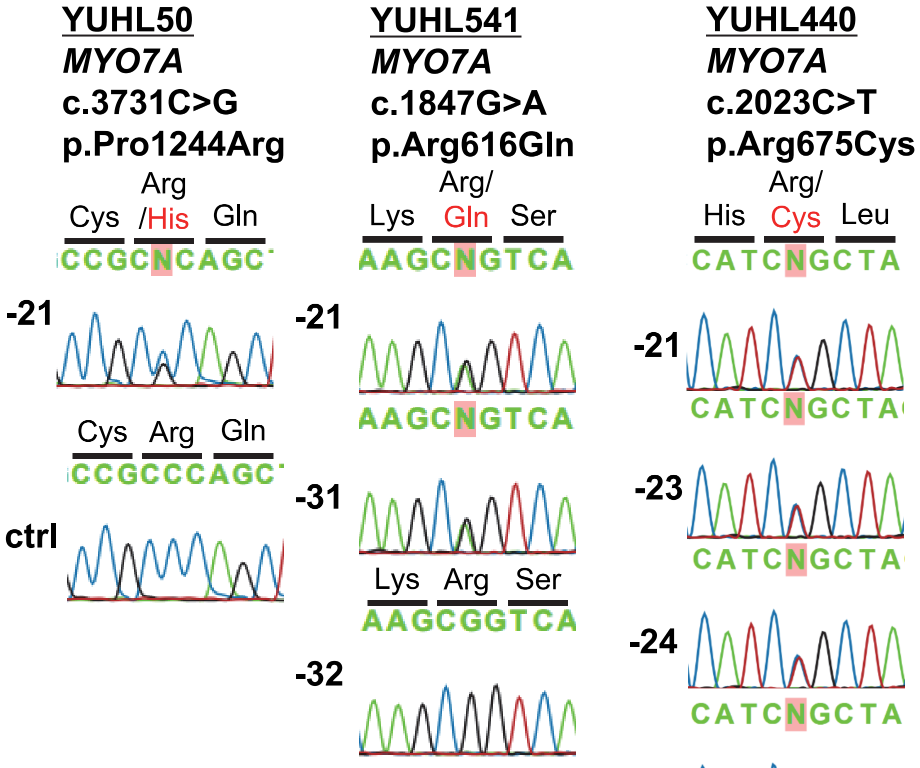

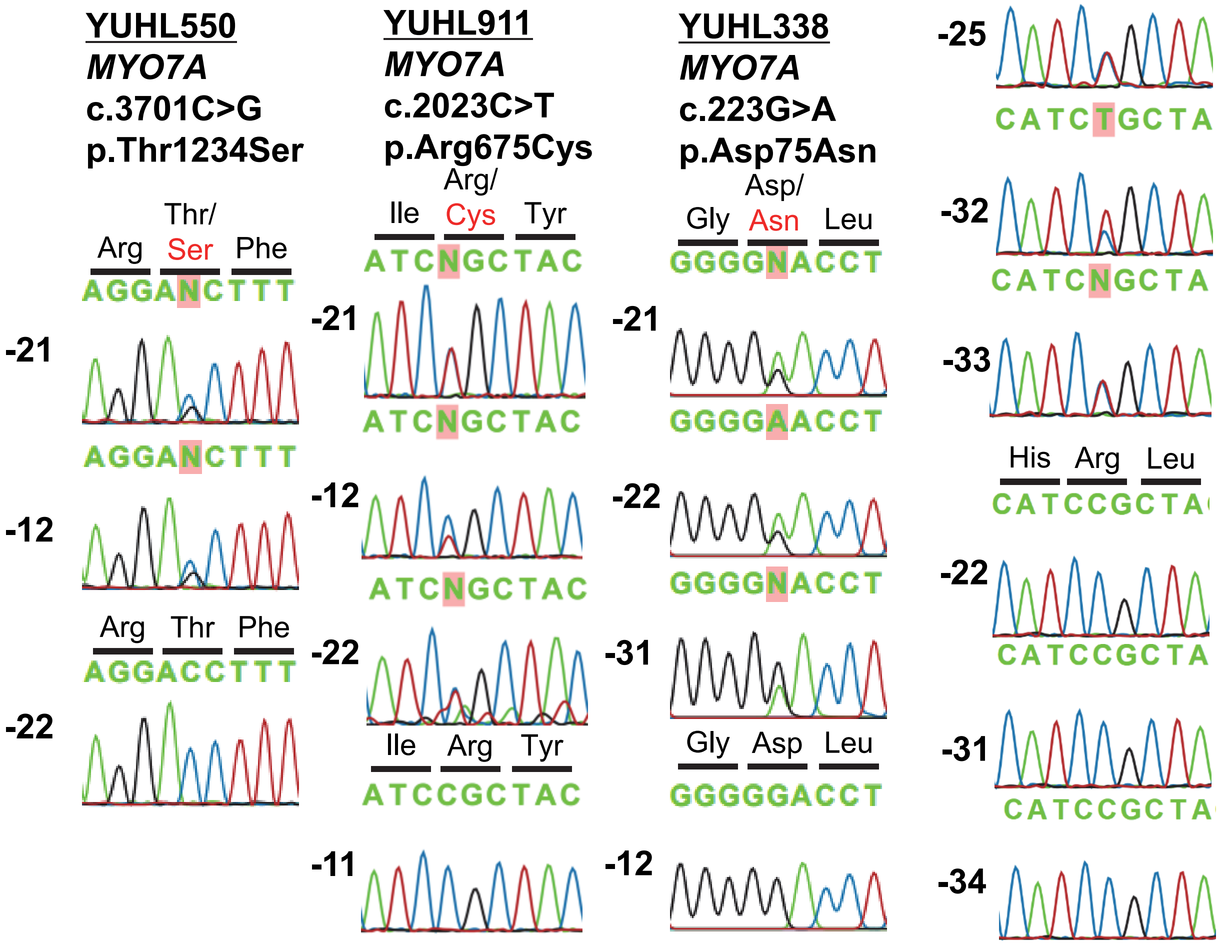

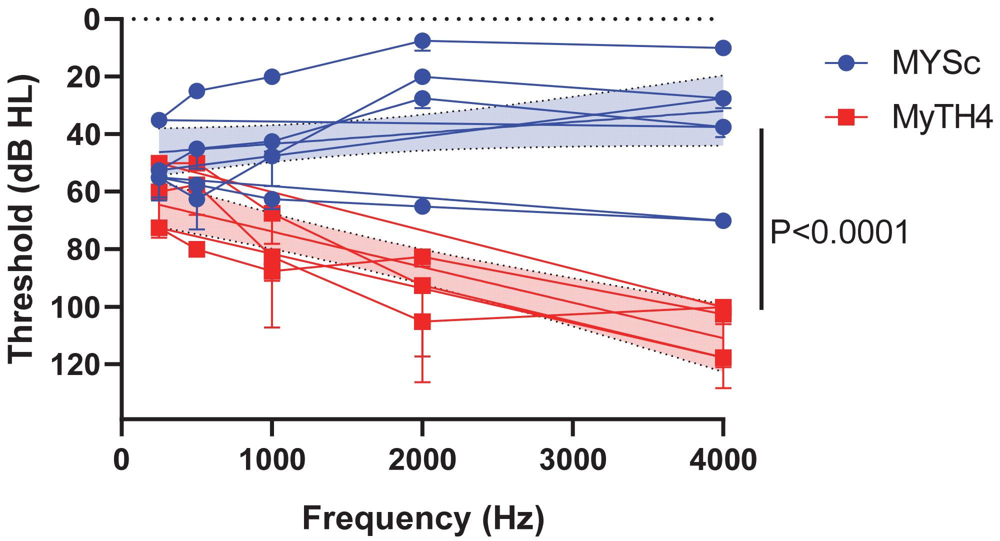

Figure S4. Audiograms of the eight affected individuals with *MYO7A* variants in YUHL cohort. The colored lines represent the pure tone thresholds of each individuals. eight of 17 affected individuals were available for their audiological phenotypes. Blue indicates PTA of patients with MYSc variants and red with MyTH4 variants. The shaded area indicates the 95% confidence interval of linear regression models fitted on each data. Comparison of slopes was made using Prism v8. The differences between the slopes are extremely significant, showing phenotypic discrepancy in YUHL cohort depends on domains wherein DFNA11 variants locate. ($p < 0.0001$)

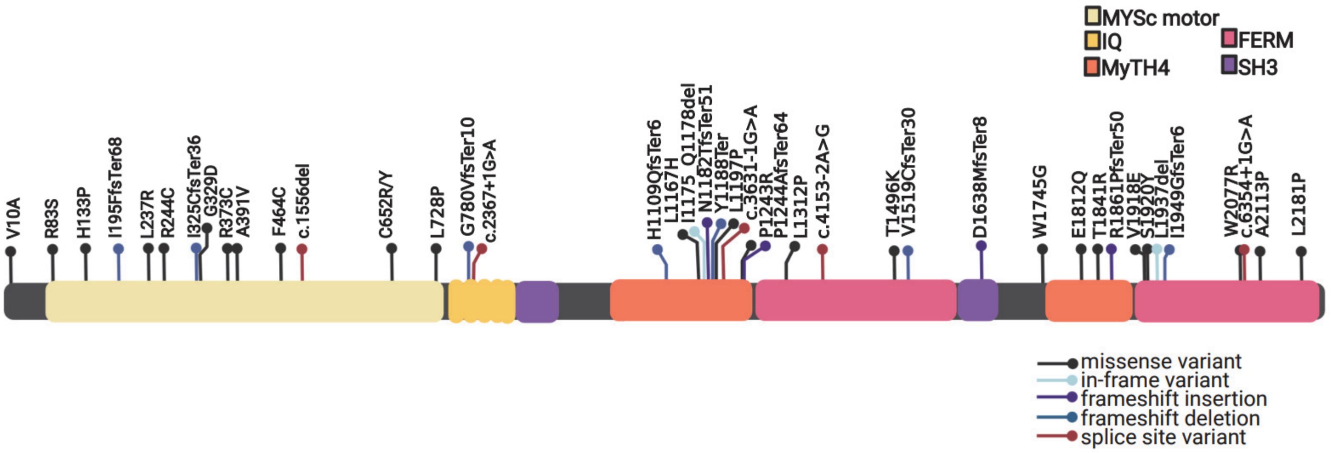

Figure S5. Distribution of DFNB2-related pathogenic *MYO7A* variants. Distribution of DFNB2-related variants satisfying AD threshold. Total of 43 variants listed as pathogenic in Deafness Variation Database.
